# RNA-Seq profile of flavescence dorée phytoplasma in grapevine

**DOI:** 10.1186/1471-2164-15-1088

**Published:** 2014-12-11

**Authors:** Simona Abbà, Luciana Galetto, Patricia Carle, Sébastien Carrère, Massimo Delledonne, Xavier Foissac, Sabrina Palmano, Flavio Veratti, Cristina Marzachì

**Affiliations:** Istituto per la Protezione Sostenibile delle Piante, IPSP-CNR, Strada delle Cacce 73, I-10135 Torino, Italy; INRA, UMR1332 Biologie du Fruit et Pathologie, 71 avenue Edouard Bourlaux, CS20032, F-33882 Villenave d’Ornon, Cedex, France; Université de Bordeaux, UMR1332 Biologie du Fruit et Pathologie, 71 avenue Edouard Bourlaux, CS20032, F-33882 Villenave d’Ornon, Cedex, France; INRA, Laboratoire des Interactions Plantes-Microorganismes (LIPM), UMR441, Castanet-Tolosan, F-31326 France; CNRS, Laboratoire des Interactions Plantes-Microorganismes (LIPM), UMR2594, Castanet-Tolosan, F-31326 France; Dipartimento di Biotecnologie, Università degli Studi di Verona, Strada le Grazie 15, I-37134 Verona, Italy

**Keywords:** Flavescence dorée phytoplasma, RNA-Seq, *Vitis vinifera*, Group II intron, Hypothetical proteins, qRT-PCR

## Abstract

**Background:**

The phytoplasma-borne disease flavescence dorée is still a threat to European viticulture, despite mandatory control measures and prophylaxis against the leafhopper vector. Given the economic importance of grapevine, it is essential to find alternative strategies to contain the spread, in order to possibly reduce the current use of harmful insecticides. Further studies of the pathogen, the vector and the mechanisms of phytoplasma-host interactions could improve our understanding of the disease. In this work, RNA-Seq technology followed by three *de novo* assembly strategies was used to provide the first comprehensive transcriptomics landscape of flavescence dorée phytoplasma (FD) infecting field-grown *Vitis vinifera* leaves.

**Results:**

With an average of 8300 FD-mapped reads per library, we assembled 347 sequences, corresponding to 215 annotated genes, and identified 10 previously unannotated genes, 15 polycistronic transcripts and three genes supposedly localized in the gaps of the FD92 draft genome. Furthermore, we improved the annotation of 44 genes with the addition of 5′/3′ untranslated regions. Functional classification revealed that the most expressed genes were either related to translation and protein biosynthesis or hypothetical proteins with unknown function. Some of these hypothetical proteins were predicted to be secreted, so they could be bacterial effectors with a potential role in modulating the interaction with the host plant. Interestingly, qRT-PCR validation of the RNA-Seq expression values confirmed that a group II intron represented the FD genomic region with the highest expression during grapevine infection. This mobile element may contribute to the genomic plasticity that is necessary for the phytoplasma to increase its fitness and endorse host-adaptive strategies.

**Conclusions:**

The RNA-Seq technology was successfully applied for the first time to analyse the FD global transcriptome profile during grapevine infection. Our results provided new insights into the transcriptional organization and gene structure of FD. This may represent the starting point for the application of high-throughput sequencing technologies to study differential expression in FD and in other phytoplasmas with an unprecedented resolution.

**Electronic supplementary material:**

The online version of this article (doi:10.1186/1471-2164-15-1088) contains supplementary material, which is available to authorized users.

## Background

Grapevine flavescence dorée phytoplasma (FD) is one of the most severely damaging diseases affecting European vineyards. This quarantine pest continues to have a significant economic impact not only in the two major European wine-producing countries, Italy and France, but also in Spain, Switzerland, Portugal, Austria, Croatia, Slovenia, Serbia and Hungary [1]. The cicadellid leafhopper *Scaphoideus titanus* Ball is the only known vector that transmits the disease from grapevine to grapevine [2]. Symptoms appear in early summer and increase in incidence and severity until harvest. These symptoms consist of drying of berry peduncles and consecutive shrivelling or drying of berries, leaf discolouration and downward leaf curling [3]. Consequently, grapevine vitality and yields are reduced, and the production of wine is irreparably compromised.

Phylogenetical analyses indicated that FD can be divided into two taxonomic 16S groups, 16SrV-C and 16SrV-D [4, 5], and three genetic clusters according to the sequence of the *map* gene: map-FD1 (including isolate FD70), map-FD2 (including isolates FD92 and FD-D) and map-FD3 (including isolate FD-C) [6]. The three clusters show different geographical distributions. In Piedmont, which is one of the most renowned Italian wine-making regions, both FD-C and FD-D isolates have been detected [7]. In this region, the local administration spent nearly € 1.5 million per year from 1999 to 2003 in disease control programs. Additionally, in 2005 the Italian government and the European Union spent € 34 million to refund growers for yield losses and replanting [8]. Better knowledge of the molecular interaction between the pathogen and its hosts is thus essential to develop new and sustainable control strategies to reduce the non-target impacts of the compulsory insecticide treatments against the vector.

New high-throughput “omics” technologies, such as whole transcriptome sequencing (RNA-Seq) and high-resolution mass spectrometry, allow simultaneous examination of thousands of genes, transcripts, proteins, and metabolites, opening new possibilities towards marker discovery and genome-wide identification of signalling molecules, protein functions and interactions. Such technologies were recently applied also to the study of plant-phytoplasma interactions, but in most of these cases only the plant responses to the phytoplasma infection have been investigated, leaving the phytoplasma perspective almost unexplored [9–16]. Only two reports took advantage of the new high-throughput technologies to describe the transcriptional and proteome landscape of phytoplasmas: Ji and colleagues provided the first valuable dataset of mulberry dwarf phytoplasma proteins by a shotgun proteomics approach [17] and, more recently, Siewert and colleagues [18] combined RNA-Seq and shotgun proteomics to provide insights into the expressed genes of ‘*Candidatus* Phytoplasma mali’ infecting graft-inoculated *Nicotiana occidentalis* leaves. Microarrays were instead used for the first global gene expression study on phytoplasmas, which evaluated the changes in gene expression during ‘*Candidatus* Phytoplasma asteris’ “host-switching” between plant to insect hosts [19].

In the present study, RNA-Seq provided the first comprehensive transcriptomics landscape of FD phytoplasma infecting field-grown cv. Barbera grapevines. An annotated draft genome of the FD92 isolate, covering 85% of the 671 kbp chromosome, has been recently produced using a combination of 454 pyrosequencing and Illumina/Solexa [20, 21]. In the absence of a complete FD genome sequence, different approaches to separate FD-mapping reads from the eukaryotic ones followed by assemblies and recursive merging of sequence datasets were used to reconstruct the FD transcripts. In this way, previously unannotated regions, polycistronic transcripts, 5′/3′ UTR regions and non-coding RNAs (ncRNAs) have been identified. Moreover, genes potentially involved in plant-phytoplasma interactions were selected to validate the RNA-Seq results by qRT-PCR on field-grown grapevines infected by either 16SrV-C or -D isolates.

## Results

### Phytoplasma detection, strain characterization and quantification

Diagnostic assays confirmed the presence of FD and the absence of stolbur ‘Bois noir’ (BN) phytoplasma in all three grapevine leaf samples used in this study (120, B68 and B75). In addition, qRT-PCR assays on the most common viruses reported in Piedmont vineyards revealed the presence of Grapevine rupestris stem pitting-associated virus in all samples and Grapevine fleck virus in sample 120 and B75.

On the basis of the *Taq*I-RFLP profiles of the ribosomal 16S gene, samples 120 and B68 were identified as 16SrV-C isolates and sample B75 as a 16SrV-D isolate (data not shown). In the three samples, the phytoplasma titer expressed as number of FD cells/500 mg of plant tissue ranged from 5.81E+05 to 6.32E+06.

### Relative quantification of grapevine and phytoplasma mRNAs

The ratio between the grapevine and the phytoplasma transcripts was evaluated by qRT-PCR, considering the expression of two single-copy genes: FD *sec*Y and grapevine ubiquitin. The transcriptional levels of these two genes were estimated to be 6 and 32000 copy number, respectively, so FD transcripts represented nearly 0.02% of the total grapevine messengers.

### Data coverage

Total RNA extracted from sample 120 was split in two parts, named sample 120 and sample 120E.

The two samples were analysed separately as technical replicates in independent sequencing runs, generating two sets of total FD transcriptome profiles.

A total of 125,813,174 and 129,412,231 paired-end (PE) reads (2x100bp) with an average insert size of 153 bp ± 39 bp was generated from libraries 120 and 120E, respectively.

The quality control and trimming resulted in two filtered sets (95% of reads passed the control) assembled with three different strategies. The effectiveness of each assembly approach was evaluated and compared to the others in order to choose the best performer and obtain the best description of the FD transcriptome (Additional file 1).

### Reads assembly

As a first assembly approach, reads from libraries 120 and 120E were first mapped to the FD92 draft genome (Table 1) and then assembled. The number of PE reads mapping in the antisense orientation to the FD92 predicted coding sequences (Table 1, third row) was below the theoretical maximum number of spurious cDNAs generated during the second-strand synthesis of the reverse transcription (calculated to be 145 reads for library 120E and 189 for library 120), so they were not further analyzed.

**Table 1:** **Number of PE reads from libraries 120E and 120 that mapped at least once to the coding and non-coding regions of the FD92 genome**

	120E	120	Total (120E + 120)
**PE Reads mapping concordantly to the FD92 rRNA/tRNA molecules**	1864	4026	5890
**PE reads mapping concordantly to the FD92 annotated CDSs in the sense orientation**	2891	3068	5959
**PE reads mapping concordantly to the FD92 annotated CDSs in the antisense orientation**	99	62	161
**PE reads mapping concordantly to the FD92 IGRs**	2431	2323	4754
**PE reads mapping concordantly to the FD92 genome**	7285	9479	16764

In each library nearly 0.01% of the total reads were mapped, confirming the ratio obtained by qRT-PCR. The two separate assemblies obtained from the libraries (dataset 1 and dataset 2) were compared to evaluate the technical reproducibility. Some sequences were unique to each dataset, i.e. they did not find any significant hits in others, whereas many other sequences found at least one significant hit with 100% identity across the alignment, but with a different length. These results suggested the importance of dealing with at least two technical replicates when studying the genome-wide transcriptome profile of an organism whose sequences represent a very small fraction of the total RNA-Seq library. A merged dataset (dataset 3) was obtained from the assembly of these two initial datasets.

The second assembly approach consisted in merging reads from the two libraries, mapping them to the FD92 genome and then assembling them altogether. The resulting dataset (dataset 4) was compared to dataset 3 to determine the best assembly strategy. Sequences from dataset 4 included all the sequences of dataset 3 and were generally longer than the corresponding sequences of dataset 3. Therefore, the second assembly approach proved to be far more efficient than the first one. A new merged dataset (dataset 5) was obtained from the assembly of dataset 3 and 4.

As a final approach, reads from the two merged libraries were first mapped to the *V. vinifera* genome. The *Vitis*-unmapped reads, which represented nearly 0.7% of each library, were then *de novo* assembled. The resulting sequences with significant similarities to known Mollicutes genes (dataset 6) were compared to dataset 5. No unique sequences were identified in dataset 6, but still there were differences in length between some matching sequences from the two datasets. The third assembly approach was found to be less efficient than the second one, both in terms of number and in terms of the average length of the assembled sequences. However, it was adopted in combination with the second approach to obtain a more accurate description of the FD transcriptome.

A final comprehensive FD transcriptome dataset of 347 sequences with an average length of 294 bp was created by assembling dataset 5 and 6 and used for further analyses.

### Comparison of RNA-Seq transcripts to FD92 annotated genes

334 out of 347 RNA-Seq sequences corresponded to 215 FD92 annotated genes (18 were full-length protein-coding transcripts) (Additional file 2). Of the remaining 13 sequences, 10 were present in the FD92 genome but the matching regions were not yet annotated, and three did not find any match to the FD92 genome but showed significant similarities to Poinsettia branch-inducing phytoplasma sequences (Additional file 2). PCR analyses with specific primers demonstrated that these three sequences truly belonged to the phytoplasma genome (Additional file 3), so they were probably localized in the gaps of the FD92 draft genome.

The 10 unannotated transcripts (Additional file 2) were further analyzed to determine whether they showed significant similarities to known Mollicutes protein-coding sequences or previously characterized ncRNAs. After querying the NCBI “nr” and the Rfam databases, four of them showed significant similarities to three phytoplasma hypothetical proteins and a translation initiation factor IF-3. In addition, contig12 and contig6 showed significant similarities (expected value, E-value ≤ 1E-5) to ncRNAs, namely the catalytically active RNA of a group II intron and the RNA component of a bacterial ribonuclease (RNase) P class B, respectively. The remaining four unannotated transcripts showed putative ORFs spanning the whole length or a part of the nucleotide sequence, but the corresponding predicted proteins did not have any significant similarity to other bacterial sequences.

RNA-Seq data also provided the opportunity to extend the length of some transcripts compared to the automatic computer annotation of FD92 genes: 34 sequences were extended by at least 1 nucleotide upstream of the predicted translation start (adding a potential 5′ untranslated region – 5′ UTR) and 11 sequences were extended downstream of the predicted stop codon in 11 sequences (adding a potential 3′ UTR) (Additional file 2). The gene coding for the 30S ribosomal subunit protein S8 *rps*H was extended in both directions.

Transcripts that i) mapped to IGRs between two non-overlapping consecutive CDS on the same strand and ii) partially overlapped the two genes on the same strand were considered as parts of polycistronic transcripts (Additional file 2, see FD92 annotated transcripts with two hits). According to our data, there were at least 15 polycistronic transcripts in FD transcriptome, most of them involving ribosomal proteins. Excluding operons that were made up only of ribosomal transcripts, most polycistronic transcripts contained two or three genes (Table 2).

**Table 2:** **Polycistronic transcripts as identified by the RNA-seq assembly**

Number of genes in operon	Gene 1	Gene 2	Gene 3
2	fba-flado_0482_0037	Cof-flado_0482_0038	
2	pdhB-flado_8220_0009	Ctg8220_0011015_0011245_f2-flado_8220_0010	
3	grpE-flado_0031_0008	dnaK-flado_0031_0007	dnaJ-flado_0031_0006
2	Ctg8084_0025861_0026808_r1-flado_8084_0018	mnmE-flado_8084_0017	
3	rpsG-flado_6343_0020	fusA-flado_6343_0021	tuf-flado_6343_0022
2	tdk-flado_6333_0005	tadA-flado_6333_0004	
2	trmD-flado_0314_2012	rplS-flado_0314_0009	
2	hup-flado_0234_0030	rpmG-flado_0234_0031	

Genes are reported in order, starting from the 5′ of the polycistronic transcript.

As sample 120 showed a 16SrV-C restriction profile whereas FD92 is a 16SrV-D isolate [6], polymorphisms identified at the nucleotide level in the comparison between RNA-Seq data and the FD92 genome could be exploited for isolate genotyping (Additional file 4).

### Phytoplasma *in silico*gene expression

The expression levels of a dataset that included the FD92 annotated genes extended by the newly identified 5′ UTRs and 3′ UTRs and the newly identified transcripts, both coding and non-coding, are shown in Additional file 5. The most expressed transcript, named contig12, corresponded to the catalytic RNA component of a group II intron, which was present in the FD92 genome in at least two slightly divergent copies. It is important to underline that one of these two copies was artificially truncated due to its position at the end of a genomic contig. Even if divided by two, the transcriptional level of this catalytic intron remained the highest in both RNA-Seq libraries. Interestingly, a blastx analysis of contig12 revealed the presence, at the 3′ end of the sequence, of the first 27 amino acids of a putative transposase tra5 for insertion sequence element IS150 (GenBank:WP_015637618.1). A further analysis of the complete copy corresponding to contig12 revealed that it did not contain any internal protein-encoding open reading frames, so it supposedly belonged to the category of the group II “ORF-less” introns. No sequences with significant similarity to *ltr*A, an intron II reverse transcriptase already annotated in other phytoplasmas genomes, were found either in the RNA-Seq data or in other regions of the FD92 genome. Additionally, the *tra5* fragment identified in contig12 was truncated not only in the transcript but also in the corresponding genome region.

The first 20 most expressed transcripts included also the immunodominant protein Imp, the variable membrane protein VmpA, the ribozyme component of a bacterial Rnase P class B, six hypothetical proteins, the GroEL chaperonin and nine proteins involved in the translation process (Table 3).

**Table 3:** **The top 20 highly expressed FD genes during** ***V. vinifera*** **infection**

locus ID	Description	RPK (mean)
Contig12	Group II catalytic intron	2749.2
Ctg0426_0019664_0020134_f2_flado_0426_0012	Imp	889.1
Ctg0234_0028748_0029098_f2_flado_0234_0025	Hypothetical protein with cold-shock binding domain	548.8
Ctg0482_0000001_0000355_r3_flado_0482_0001	Hypothetical protein with 1 TMD + SP	460.8
rplP-flado_0067_0029	50S ribosomal subunit protein L16	95.6
tuf-flado_6343_0022	Elongation factor EF-Tu	95.6
Contig6	Bacterial Rnase P class B	80.3
Ctg7221_0018383_0018643_r1_flado_7221_0014	Hypothetical protein with 1 TMD + SP	54.7
Ctg5304_0020454_0020660_f3_flado_5304_0019	Hypothetical protein with 1 TMD	48.6
vmpA-flado_0482_0026	Variable membrane protein A	48.3
Ctg7221_0002401_0002754_f1_flado_7221_0002	Hypothetical protein with 1 TMD + SP	47.9
rpsU-flado_5304_0006	30S ribosomal subunit protein S21	46.0
rplV-flado_0067_0031	50S ribosomal subunit protein L22	43.9
rpsK-flado_0067_0011	30S ribosomal subunit protein S11	41.8
Ctg0314_0004067_0004231_f2_flado_0314_0005	Hypothetical protein	41.7
groL-flado_0426_0003	Chaperonin GroEL	40.3
rplN-flado_0067_0026	50S ribosomal subunit protein L14	37.1
rpsM-flado_0067_0012	30S ribosomal subunit protein S13	37.1
fusA-flado_6343_0021	Elongation factor EF-G	34.5
rplB-flado_0067_0033	50S ribosomal subunit protein L2	33.4

Transcription levels are expressed as the mean of RPK (reads per kilobase of transcript) values obtained in the two expression libraries. The first column (locus ID) reported the IDs of the annotated FD92 genes, with the only exception of contig12, which was obtained in this study. A brief gene description and the mean of reads per kilobase of transcript (RPK) are also reported. TMD = transmembrane domain predicted by TMHMM; SP = signal peptide predicted by SignalP-HMM v. 3.

**Figure 1:**
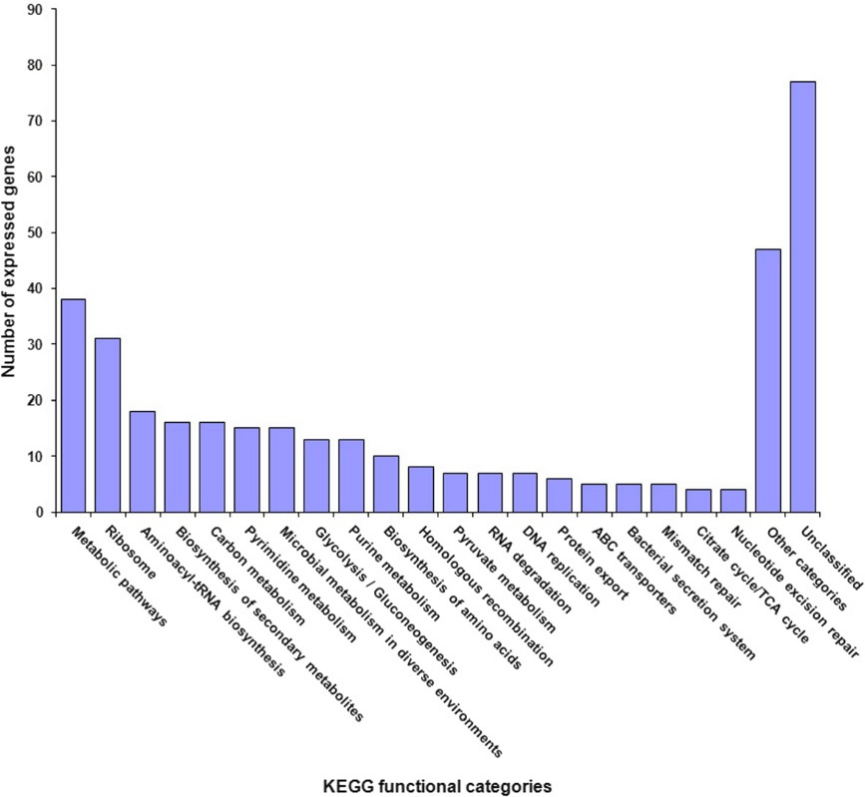
**Distribution of assembled transcripts into functional categories according to KEGG classification.** Number of expressed genes (y-axis) associated to KEGG pathways (x-axis).

Expressed transcripts were classified by comparing sequences against the manually curated KEGG GENES database (Additional file 5). Excluding the generic functional category named “Metabolic pathways”, the most represented categories during *Vitis* infection were those related to translation and protein metabolism (ribosome, tRNA and amino acids biosynthesis), DNA metabolism (pyrimidine and purine metabolism, DNA replication, DNA repair) and carbon metabolism (including glycolysis/gluconeogenesis) (Figure 1). In particular, all the enzymes of the glycolysis pathway were transcribed. Transport, protein export and secretion categories were also well represented with a total of 16 transcripts. Unfortunately, nearly 30% of the total assembled transcripts were not functionally classified.

**Figure 2:**
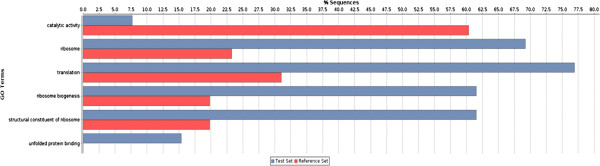
**Enrichment analysis of GO terms calculated by Fisher’s exact test.** Reference set (red) is represented by all the FD expressed genes, whereas the test set (blue) is represented by the top 20 highly expressed FD genes.

Where possible, protein-coding transcripts were associated to GO terms by Blast2GO (Additional file 5). In order to identify whether any GO term was over-/under-represented in the list of the 20 most expressed transcripts, an Enrichment Analysis with two-tailed Fisher’s exact test *(*P < 0.05) was performed against all the expressed genes. The over-represented GO terms were related to translation and protein metabolism (“structural constituent of ribosome”, “ribosome biogenesis”, “translation”, “unfolded protein binding”, “ribosome”), whereas the only under-represented GO term was “catalytic activity” (Figure 2 and Additional file 5).

### Validation of RNA-Seq expression values by qRT-PCR

Target genes for qRT-PCR assays were selected taking into account three criteria: 1) a wide range of *in silico* expression levels, from the highest (contig12 with RPK = 2749) to the lowest (*rpo*D with RPK = 0), 2) the possible involvement in the host-phytoplasma interactions and 3) the annotation as “hypothetical proteins” of unknown function. Fifteen target genes were selected: contig12 as the most expressed transcript, two known surface-exposed proteins (Imp and VmpB) [22–25], five hypothetical proteins (comp83, comp115, comp100, comp126, PAM266), a protein known to regulate the excretion of virulence factors in other bacteria (SpoVG) [26], a protein involved in the secretion pathway (ftsY), two proteolytic enzymes potentially contributing to virulence (ysdC and tldD), a protein involved in the defence mechanisms against oxidative stress (osm), the RNA polymerase sigma factor rpoD and an ABC transporter (CoABC) (Table 4).

**Table 4:** **Transcripts selected for validation by qRT-PCR**

locus ID	Abbreviation	Description	RPK (mean)	EI (mean)
Contig12	contig12	Group II intron	2749.2	10.9
Ctg0426_0019664_0020134_f2_flado_0426_0012	Imp	Imp	889.1	3.5
Ctg0234_0028748_0029098_f2_flado_0234_0025	comp83	Hypothetical protein with cold-shock binding domain	548.8	2.1
Ctg7221_0018383_0018643_r1_flado_7221_0014	comp100	Hypothetical protein	54.7	1.1
Ctg0314_0004067_0004231_f2_flado_0314_0005	comp115	Hypothetical protein with 1 TMD + SP	41.7	1.4
Ctg0482_0033346_0033975_f1_flado_0482_0023	spoVG	septation protein	32	0.5
ysdC-flado_4539_0017	ysdC	β-glucanase	29.2	0.5
Ctg0338_0006451_0007086_f1_flado_0338_0005	comp126	Hypothetical protein with 1 TMD + SP	23.3	0
vmpB-flado_0031_0011	vmpB	Variable membrane protein B	13.1	0.1
tldD-flado_0426_0020	tldD	Metalloprotease	12.2	0.2
ftsY-flado_0482_0016	fstY	Signal recognition particle receptor	9.1	0.1
Ctg0314_0007223_0007996_f2_flado_0314_0008	PAM266	Hypothetical protein (similar to PAM266)	8	0.1
CbiO2-flado_0067_0008	CoABC	Cobalt transporter ATP-binding subunit	5	0.2
Ctg5304_0021069_0021488_f3_flado_5304_0021	osm	OsmC-like protein	4.8	0.1
rpoD-flado_4539_0007	rpoD	RNA polymerase sigma factor	0	0.5

Selected genes are listed according to the IDs of the FD92 annotated genes (locus ID), with the only exception of contig12, which was obtained in this study. The abbreviation used in this work, a brief gene description, the mean of reads per kilobase of transcript (RPK) and the mean of the expression indices (EI, N° of transcript per FD cell) are reported.

The existence of transcripts corresponding to contig12 and to the hypothetical proteins was first confirmed by RT-PCR on the same RNA used for the RNA-seq library construction (data not shown). Expression of the selected FD genes was examined in three FD-infected grapevine samples: sample 120, B68 and B75. To estimate the expression level of the selected FD transcripts, the mRNA absolute quantity was compared to the phytoplasma titer measured in the corresponding sample. The primers list, the corresponding amplification conditions and efficiencies, melting peak temperatures and correlation coefficients of qRT-PCR reactions were reported in Additional file 6.

**Figure 3:**
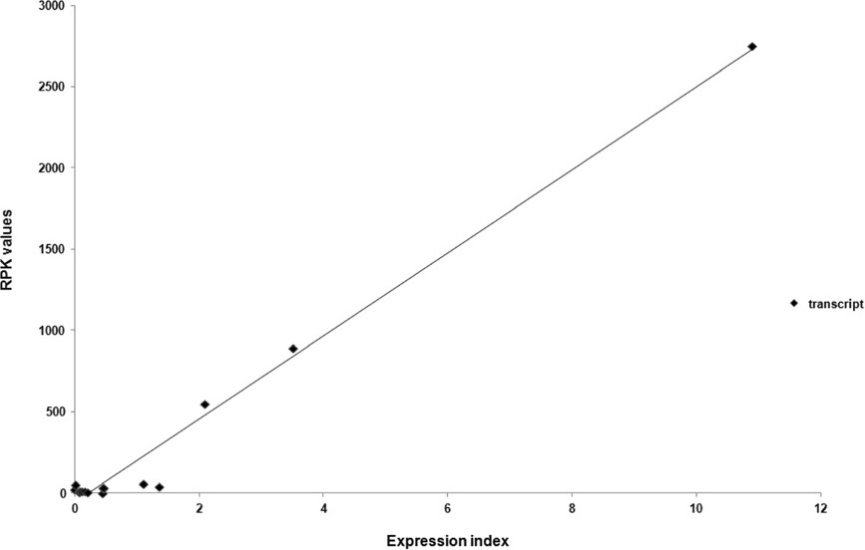
**Correlation between RNA-Seq and qRT-PCR expression data.** Plot of the transcriptional levels of the 15 selected genes expressed as RPK values (y-axis) and Expression Index values (x-axis). R^2^ = 0.98 (P < 0.05).

For each gene, the average Expression Index (EI, N° of transcript in single FD cells) in the three grapevine samples was calculated (Table 4 and Additional file 5). The reliability and reproducibility of the expression values of the selected transcripts were confirmed by the squared correlation coefficient (r2 = 0.98) calculated between the qRT-PCR and the RPK values (Figure 3).

According to the Welch-one way test, the mean of expression levels was significantly different among target transcripts (P < 0.05). Contig12 was the most abundant transcript in all three grapevine samples, irrespective of their restriction profiles, with an average EI significantly higher than all the other genes.

## Discussion

Application of deep sequencing technologies to specifically study the transcriptome of intracellular obligate endosymbiont bacteria has been attempted in very few cases, because the selection of prokaryotic transcripts in a multitude of eukaryotic RNAs makes the accurate representation of the bacterial transcriptome particularly challenging. To our knowledge, the only overall gene expression studies conducted so far on phytoplasmas was that of Siewert and colleagues [18] on ‘Ca. P. mali’. Other whole-transcriptome analyses of obligate intracellular bacteria were, for instance, concerned with *A. phagocytophilum* [27], *Lawsonia intracellularis* [28] and *Wolbachia* in symbiosis with *Onchocerca volvulus* [29]. In almost all of these studies, including the one on ‘Ca. P. mali’ , hosts were artificially infected and kept in laboratory conditions. The study on *Wolbachia* was so far the only one in which an obligate intracellular bacterium was analyzed under natural conditions, i.e. in nematodes living in cattle raised in field. In all cases, a bacterium-enrichment procedure was undertaken by selecting particular cell types/tissues and/or by adopting specific experimental procedures (selective hybridization, host rRNA depletion or polyA transcript depletion)*.* In our work, samples were collected in field from a Piedmontese vineyard in late July and the pathogen-enrichment process consisted of coupling the RNA extraction from leaf midribs to eukaryotic rRNA depletion.

In ‘Ca. P. mali’ , the RNA-Seq approach resulted in 468 mapped reads, corresponding to 132 expressed genes [18]. In our study, deep sequencing of a FD-infected grapevine sample followed by three assembly approaches allowed us to obtain an average of 8300 FD-mapped reads per library, which represented a better coverage of the reference transcriptome. As in many other genomic-scale transcriptomics surveys, also in this study RNA-Seq proved to be an extremely powerful technique for a) the detection and quantification of previously unannotated transcripts, b) the discovery of the polycistronic organization of some transcripts and c) the addition of 5′/3′ UTRs to protein-coding genes. This kind of data becomes extremely precious especially when dealing with obligate intracellular bacteria, which can neither be grown under laboratory conditions nor genetically manipulated.

In our work, we assembled 347 sequences corresponding to 219 protein-coding genes (4 were previously unannotated) and we found that 273 FD92 genes showed at least one mapped read in both libraries. Mastronunzio and colleagues identified 82 *A. phagocytophilum* expressed genes [27], whereas Vannucci and colleagues reported that 754 protein-coding genes of *L. intracellularis* showed at least one mapped read [28]. Our results are close to the number of expressed transcripts reported for other obligate intracellular bacteria*,* despite the fact that the number of reads mapping to those genomes was higher, ranging from hundreds of thousands to millions of reads.

Analyzing global transcriptional levels, genes related to ribosomal structure and biogenesis, transcription and protein biosynthesis were the most expressed in FD, as well as in *L. intracellularis* [28]. This result, along with the absence of highly expressed genes related to the DNA replication machinery or cell cycle, suggests that, in late July, when the FD titer has been demonstrated to be the highest [30], phytoplasma cells had already undergone extensive cycles of replication and growth. Therefore, most of the energy could be rerouted to protein synthesis. Interestingly, the chaperone GroEL was included in the list of the FD most expressed genes. Its remarkable ability to rescue non-productive protein conformations is particularly crucial in obligate endosymbiotic bacteria, in which the fixation of slightly deleterious mutations as a result of their distinctive lifestyle often affects the functional conformation of proteins [31].

Excluding the transcription/translation-related proteins, almost all the other proteins in the list of FD top 20 highly expressed genes are known or predicted membrane proteins. Imp and Vmp proteins are well known cell-surface proteins and several studies suggested they might have a prominent role in the host–phytoplasma interaction [22–25]. In addition, two hypothetical proteins (Ctg0482_0000001_0000355_r3_flado_0482_0001 and Ctg7221_0018383_0018643_r1_flado_7221_0014) were predicted to have one transmembrane region and a cleavage site, so they are likely to remain attached to the FD membrane after secretion. The nucleotide polymorphisms we observed in some membrane protein-coding genes between the FD92 genome and our RNA-Seq data are consistent with the necessary adaptations of the bacterium to its complex and changing relationship with the host. These differences could be exploited for studying the FD genetic diversity within different strains.

Besides genes related to protein synthesis, the functional classification highlighted a high percentage of unclassified genes. This was probably due to a) the lack of a good functional annotation for some known phytoplasma proteins, such as SpoVG, Imp, VmpA, VmpB, etc.…, and b) the presence of many hypothetical proteins whose functions are still unknown and thus not yet included in databases.

It is noteworthy that the abundance of hypothetical proteins (six in total) among the FD most expressed genes is in accordance to the results obtained in *L. intracellularis* [28] and *Wolbachia* [29], in which seven and four out of the 20 most expressed genes encoded hypothetical proteins, respectively. Even in ‘Ca. P. mali’ the highest number of RNA-Seq reads was assigned to a conserved hypothetical integral membrane protein [18]. Therefore, such hypothetical proteins of unknown function should be the main targets for future analyses to elucidate their potential role in host-bacterial interactions.

Other known potential phytoplasma effectors, such as SAP-like proteins [32–34], HflB proteases and AAA+ ATPases [35], superoxide dismutases [36], proteins of the *Sec*-dependent secretion pathway [37], were expressed, but not listed among the most expressed genes in FD.

The most striking results were the high expression levels of two ribozymes that were not previously annotated in the FD92 genome: the RNA component of a bacterial RNase P class B and the catalytic RNA of a group II intron. Bacterial RNases P are made up of two components: a catalytic RNA and a polypeptide chain. Together they function as an endoribonuclease that removes the precursor sequence from the 5′ end of a primary tRNA transcript to generate mature tRNAs [38]. The high level of transcription of the RNA component of this ribonucleoprotein complex was consistent with the high expression of genes related to translation, as tRNA maturation is an essential process for protein biosynthesis.

Widespread in prokaryotes and in organelles of fungi, plants and lower eukaryotes, group II introns are genetic retroelement capable of self-splicing and inserting into DNA sites [39]. They typically consist of a ribozyme, which catalyzes splicing events, and a protein, which takes part both in splicing and insertion events. In prokaryotes, the protein component, which shows endonuclease, reverse transcriptase and maturase domains, is encoded by the intron itself and is essential for both retrohoming and retrotransposition [39]. In the FD92 genome two almost identical copies of this group II intron were identified so far. One of these was artificially truncated at the 3′ due to its position at the end of a genomic contig, so only the other copy was analyzed in more detail. The complete genomic copy of this group II intron was very similar (77% identity, E-value = 4E-39) to the Onion yellows phytoplasma intron named OYPI1 (GenBank:AP006628 Region: 388234–390749) [40], but, unlike OYPI1, it seemed to be an ORF-less intron, as neither a reverse transcriptase nor any other protein-coding gene was detected within the intron sequence. As already hypothesized for Onion yellows phytoplasma ORF-less OYPI2 (GenBank:AP006628 Region: 544682–545416) [41], isolate FD92 could also harbor in the gaps of its unfinished genome at least one full-length group II intron that may act in *trans* on the other ORF-less group II intron(s). It has, in fact, been shown that group II introns that became fragmented by genome rearrangements in eukaryotic organelles have the capacity to ligate independently transcribed coding sequences, splice accurately *in vivo* and finally produce a functional mRNA [42]. The presence of a *tra5* remnant at the 3′ of contig12 suggested that the absence of the retrotranscriptase in this copy could indeed be associated with some genome rearrangements. Finally, we cannot exclude that the missing part of the other copy of group II intron could encode itself a functional reverse transcriptase.

The high transcriptional levels of contig12 and the presence in the FD92 genome of other proteins usually associated with group II intron mobility, such as a complete recA and a DEAD-box protein [43, 44], may indicate that the retroelement can be fully functional.

To our knowledge, this is the first report of the expression of a phytoplasma group II intron during plant infection. The high transcriptional levels of this gene were confirmed by qRT-PCR on the same sample used for the RNA-Seq library construction (sample 120) and on other two grapevine samples (samples B68 and B75), even though they belonged to two different 16S restriction profiles. Hypothesizing that contig12 could really be part of a functional retroelement, it may contribute to the genomic plasticity that is necessary for the phytoplasma to increase its fitness and, ultimately, adapt to its host. Mobile elements, in general, are known to play key roles during the emergence of host-adaptive strategies in bacteria [45] and group II introns, in particular, have been demonstrated to be responsible of some recent genomic rearrangements in the bacterial endosymbiont *Wolbachia* [46] *.*

Fourteen additional genes, including highly expressed hypothetical proteins and genes with possible involvements in the host-bacterium interactions, were examined by qRT-PCR. The positive correlation on a linear regression model between qRT-PCR and RPK values and the high R-squared value demonstrated that RNA-Seq data properly estimated the expression levels of the selected genes.

## Conclusions

The RNA-seq technology was successfully applied for the first time to analyse the FD global transcriptome profile during grapevine infection. Our results provided new insights into the FD gene structure, transcriptional organization and expression levels with an unprecedented resolution for phytoplasmas. The low number of FD-mapped reads has not prevented us from improving the genome annotation or from providing a reliable view of the FD transcriptome, but it was a limitation when exploring the antisense transcription. The strand-specific RNA-seq could have shed some light on the FD antisense transcriptional activity, which is totally unexplored. However, because the number of reads mapping to FD annotated CDSs in the antisense orientation was lower than the possible error threshold of the technique, any consideration on this subject would have been merely speculative. A further enrichment for phytoplasma sequences would be necessary not only for this purpose, but also when comparing variable conditions, e.g. the phytoplasma transcriptome in response to the host plant and to the insect vector. A higher number of phytoplasma-mapped reads could, in fact, increase the probability of finding statistically significant differences in the expression profiles. As shown in this work, the use of at least two technical replicates per sample is also essential to provide a broader and more reliable picture of the transcriptional landscape, especially when the genome coverage is low.

## Methods

### Plant material

Leaf samples from symptomatic field-grown cv. ‘Barbera’ grapevines (growth stage: 6–7 leaves separated) were collected in Cocconato (Piedmont, Italy) in late July 2013. The vineyard, which consisted of approximately 8600 plants arranged in 76 north–south running rows, has been monitored since 2007 for phytoplasma infection, and so a detailed map of the sanitary status of the plants (healthy, infected and recovered) was available at the beginning of this study. Plants were regularly treated with fungicides and no typical symptoms of fungal diseases were observed during sampling. Molecular assays were used to detect the presence of FD and BN phytoplasmas [47]. Leaves infected only by FD were retained for further studies, whilst samples with mixed infections were excluded from this work. Among the FD-positive plants, sample 120 from row 62 was chosen for the RNA-Seq analysis.

### Nucleic acid extraction

For each samples, total RNA and DNA were extracted from 500 mg of pooled plant material. Total RNA was extracted following the protocol described by Chang and colleagues [48] and treated with RNase-free DNase I (Applied Biosystems, Foster City, CA, USA) to avoid residual DNA contamination. After nucleic acid precipitation with LiCl, the RNA-depleted supernatant was retained for total DNA extraction.

### Phytoplasma detection, strain characterization and quantification

For FD and BN diagnosis, 40 ng of DNA were used in direct PCR with universal primers P1/P7 [49]. Reaction products were used as templates in nested PCRs driven by primers R16 (V and I) F1/R1 [50].

For FD strain characterization, P1/P7 amplicons were also used as templates in nested PCRs with primers M1/B6 [4]. Nested PCR amplicons were then digested with *Taq*I endonuclease (Thermo Fisher Scientific, Walthem, MA, USA) for 1 h at 37°C and the restriction profiles visualized after electrophoresis on ethidium bromide-stained acrylamide gels.

### Preparation of strand-specific RNA-Seq libraries

Libraries construction and sequencing on the Illumina HiSeq 1000 were performed at the Centro di Genomica Funzionale, Dipartimento di Biotecnologie, Università degli Studi di Verona, Verona, Italy. The Epicentre Ribo-Zero™ Magnetic Kit (Plant Leaf) was used to remove rRNA from 3.5 micrograms of total RNA. The rRNA-depleted total RNA fractions were used to generate two directional libraries with the Illumina TruSeq Stranded Total RNA preparation kit. According to the kit manual, more than 98% of the mapped reads should return accurate strand origin information, whereas the remaining reads may represent non-specific background.

### Sequence analysis

Settings used for each of the following computer programs were specified in Additional file 7.

Before read mapping and assembly, poor quality data were filtered out by Trimmomatic v. 0.30 [51].

The partial FD92 draft genome (cluster FD2) [52] used in this work is available upon request at https://iant.toulouse.inra.fr/F.doree.

Bowtie v. 0.12.7 [53] was used to map reads to the FD92 genome as well as to the assembled transcripts for the evaluation of gene expression levels.

Tophat v. 1.4.1 [54] was preferred to Bowtie for mapping reads to the 12X *Vitis vinifera* genome [55], because it can identify splice junctions between exons.

Given the low amount of FD-mapped PE reads (thousands) compared to other projects dealing with transcriptomes of free-living bacteria (millions), we decided to retain, only for the transcriptome assembly, the single-end (SE) reads, i.e. those PE reads in which only one read of the couple passed the quality check. SE reads were not considered for the quantitative analysis. The minimum assembled contig length to be reported by Trinity assembler (Trinityrnaseq_r20131110) [56] was set to 150 bp to avoid losing short protein-coding RNAs, considering that 49–51 amino acid-long proteins are quite common in phytoplasmas (e.g. GenBank:WP_011161061.1, GenBank:NP_950971.1, GenBank:AAO61980, GenBank:AAO61987).

After each Trinity assembly, the assembled sequences were first analysed by blastn [57] against the FD92 genome. Assembled nucleotide sequences were considered as significantly similar to the FD92 genome if they showed at least 95% identity across the alignment with an E-value ≤ 1E-20. Those without a match were then analysed by blastn against the NCBI “nt database” to discard sequences belonging to plant plastidial/mitochondrial genomes or other bacterial genomes. Finally, sequences with no significant similarities to the NCBI “nt database” were analysed by blastx [57] against the NCBI “nr database” to retain only those with significant similarities (E-value ≤ 0.0001) to Mollicutes predicted proteins. This last step was used to find coding sequences that could potentially be localized in the gaps of the unfinished FD92 genome.

Sequence datasets obtained with different assembly strategies were merged using cap3 (version:4/15/05) [58]. After each cap3 assembly, sequences were again checked against the FD92 genome to avoid misassemblies.

A dataset enriched in FD-mapping PE reads was submitted to Sequence Read Archive (SRA) (http://www.ebi.ac.uk/ena/data/view/PRJEB6982).

### *In silico*phytoplasma gene expression

The expression level of each transcript was calculated by an ad-hoc Bash wrapper script calling Bowtie v. 0.12.7 and expressed as number of reads per kilobase of transcript (RPK). Given the low amount of FD-mapped reads, we decided to use RPK instead of RPKM (number of reads per kilobase per million reads mapped). RPK values were calculated using only PE reads that mapped concordantly, unambiguously and on the correct strand. To compare the RPK values of the two libraries, the RPK values obtained from library 120E were multiplied by a factor of 1.3, which took into account the different number of FD-mapped reads in the two libraries.

The expression level of each gene that was predicted to be part of a polycistronic transcript was evaluated separately. Only genes with mapped reads in both libraries were considered expressed in order to provide more accurate values of their transcriptional rates. The RPK values were thus provided as the mean of the RPK values of library 120 and library 120E.

23S and 16S ribosomal RNAs were excluded from the list of expressed genes, since they are usually the most abundant RNAs in all species.

### Prediction of signal peptides and transmembrane domains

SignalP v. 3.0 [59] with HMM method was used to predict the presence of signal peptides (minimum probability threshold = 0.9).

Transmembrane helices were predicted by TMHMM Server v. 2.0 [60].

### Functional analyses of expressed genes

KAAS server [61] based on bi-directional best hit information was used to identify the functional properties and the biological roles of expressed genes.

The Gene Ontology (GO) functional classification and the following enrichment analysis with Fisher’s Exact Test (test set: the top 20 highly expressed FD genes during *V. vinifera* infection; reference set: all the expressed FD genes during *V. vinifera* infection ) were performed by the Blast2GO suite [62].

### qPCR and qRT-PCR assays: FD titer, gene expression and mRNA relative quantification

All qPCR and qRT-PCR reactions were carried out in a CFX Connect™ Real-Time PCR Detection System (Bio-Rad) supported by the CFX Manager™ Software, version 3.0.

FD titer and gene expression analyses were performed on the same sample used for RNA-Seq (sample 120) and on two additional FD-infected samples, named B75 and B68, collected in the proximity of sample 120, from rows 75 and 68, respectively. These two samples were chosen according to their 16S restriction profiles: B68 showed the same 16S-C profile as sample 120, whilst B75 belonged to the FD-D cluster. To minimize potential variability due to environmental conditions, the three plant samples were collected on the same day.

Plants in Cocconato vineyard were regularly treated with fungicides and no typical symptoms of fungal diseases were observed during sampling. The three samples used in this work were tested by qRT-PCR for the presence of eight viruses, which are all common in Piedmont vineyards: Grapevine Virus A (GVA), Grapevine Virus B (GVB), Grapevine Fanleaf Virus (GFLV), GFkV, Grapevine Leaf-Roll-Associated Viruses (GLRaV) 1, 2 and 3 and Arabis mosaic virus (ArMV) [47, 63].

FD titer was calculated as already described by Roggia and colleagues [30] and expressed as number of FD cells/500 mg of leaf sample.

For qRT-PCR assays, cDNA was synthesized from 500 ng of total RNA using the High Capacity cDNA Reverse Transcription Kit (Applied Biosystems, Foster City, CA, USA).

External standard curves were produced using serial dilutions of plasmids carrying fragments of the 15 selected genes amplified by conventional PCR from total DNA of FD-C-infected periwinkle (Piedmont isolate). In particular, primers by Galetto and colleagues were used for the amplification of the *Imp* gene [64]. Plasmids carrying amplicons were used to set up standard curves, ranging from 10^8^ to 10^4^ transcript copy number. The number of plasmid copies per microlitre was derived from the concentration measured at the Nanodrop spectrophotometer, according to the equation described by Osborn and Smith [65].

SYBR Green-based qRT-PCR protocols were optimized and the final mix contained 25 ng of cDNA, 1 x iQ™ SYBR^®^ Green Supermix (Bio-Rad, Life Science Research, Hercules, CA, USA), 300 nM primers, sterile double distilled water to a final volume of 10 μl. Reaction conditions were as follows: 5 min at 95°C and 45 cycles of 30 sec at 95°C followed by 1 min at the optimized annealing and extension temperatures (Additional file 6). Samples were run in duplicate together with four tenfold serial dilutions (from 10^2^ to 10^8^ transcript copy number) of the corresponding standard plasmid. The complete qRT-PCR mix with total RNA and sterile distilled water instead of cDNA were used as negative controls on each plate. Melting curves were produced at the end of the PCR to assess the reaction specificity: the PCR products were heated to 95°C for 1 min, cooled at 65°C and held at that temperature for 1 min and then slowly heated back to 95°C at a rate of 0.5°C/cycle.

An Expression Index (EI) of each target phytoplasma gene was calculated for each sample as the ratio between the FD mRNA absolute quantity and the phytoplasma titer measured in the corresponding sample. The Shapiro-Wilk test and the Bartlett test revealed that the EIs of each gene in different samples were normally distributed, but did not show equal variances. The Welch-one way ANOVA for normally distributed and heteroscedastic data, followed by the Waller-Duncan test, was then used for multiple comparisons. All statistical analysis were performed with the R Stats package v 3.1.1 (http://www.R-project.org).

The same *sec*Y primers and amplification conditions used for calculating the FD titer were also used to estimate the relative abundance of phytoplasma mRNA in sample 120. In this case, the single-copy ubiquitin gene of grapevine (GenBank:FQ378362.1) was used instead of the 18S rDNA gene. Ubiquitin primers by Gutha and colleagues [66] were used to determine the transcript levels of this gene.

## Electronic supplementary material

Additional file 1: **Comparison among the three assembly strategies.** Table reports: how the two libraries were used for the assembly (separated or in combination), the name of the resulting dataset, the datasets that were compared two-by-two at the end of each assembly approach, the number of assembled sequences for each dataset, the average length of sequences belonging to each dataset, the number of unique sequences for each dataset and the name of the dataset obtained after the merging with cap3. * and § indicate that FD-mapped reads and unmapped *Vitis* reads were used, respectively. (DOC 36 KB)

Additional file 2: **Comparing RNA-Seq assembled sequences to the FD92 genome.** Tables report: RNA-Seq assembled sequences that matched to FD92 annotated genes (sheet: “FD92 annotated genes”); assembled transcripts with no match to the FD92 genome, but with significant similarities to other phytoplasmas proteins (sheet: “hits to other phytoplasmas”); RNA-Seq assembled sequences that matched to the FD92 genome, but were not annotated (sheet: “FD92 unannotated genes”). # and § indicate assembled transcripts that added either the 5′ UTR or the 3′ UTR region to previously annotated FD92 genes, respectively; * indicates genes that are part of a polycistronic transcript. (XLS 92 KB)

Additional file 3: **PCR products of the three RNA-Seq sequences with no match to the FD92 genome.** Figure shows the amplicons obtained from healthy (H) and FD-C infected (I) periwinkle maintained in laboratory conditions. M = 1 kb plus marker (Thermo Fisher Scientific, Walthem, MA, USA).The primers used for amplifications were: contig1f 5′- GCCTGATAGAAAAAAAGTAG -3′ and contig1r 5′ – TTAGGAGAAATTTCTCCTGTAT - 3′ (Annealing temperature = 59°C); contig2f 5′- GAGAATCTGTAATGTATAAGG -3′and contig2r 5′ - TCAATATCTTCAGGAGTAGG - 3′(Annealing temperature = 60°C); novitis_comp95067f 5′- TGTGGCGATAACAAGAGCAA - 3′ and novitis_comp95067r 5′- TGTGCATAACCTTATCTCCTGC -3′ (Annealing temperature = 62°C). (PPTX 133 KB)

Additional file 4: **Assembled transcripts that showed less than 100% identity at the nucleotide level with the FD92 annotated genes.** Maltose/maltodextrin-binding periplasmic protein malE is present twice in the list because two assembled transcripts corresponded to two different parts of the corresponding FD92 gene. Most differences corresponded to non-synonymous substitutions, so these polymorphisms could potentially affect the protein conformation and/or functionality. *known/predicted membrane proteins; ^§^ncRNA; TMD = transmembrane domain predicted by TMHMM. (DOCX 13 KB)

Additional file 5: **Transcriptional levels and functional classification of expressed genes.** Tables report: the locus ID (IDs of the FD92 annotated genes and the transcripts assembled in this work), the gene names, the RPK values of all the expressed genes in libraries 120 and 120E separately, the mean of the two RPK values, the GO classification, the enzymes codes (sheet: “expressed genes”); the locus ID, the abbreviations used in this work, the RPK values in libraries 120 and 12 0E, the mean of RPK values, the EI values in 120, B68 and B75 samples, and the mean of the EI values for the 15 selected genes (sheet: “selected genes RPK & EI”); the KEGG functional classification of the expressed gens, as performed by KAAS (sheet: “KEGG functional classification”); the GO Enrichment Analysis with Fisher’s exact test (P < 0.05) of the top 20 highly expressed genes**,** as performed by Blast2GO (sheet: “GO enrichment analysis”). (XLS 118 KB)

Additional file 6: **PCR/qPCR/qRT-PCR primers and amplification conditions.** Table reports primers sequences, amplification conditions and reaction parameters (for qRT-PCRs only) of all the PCR analyses carried out in this work. (XLS 1 MB)

Additional file 7: **Parameters used for bioinformatics analysis.** File reports for each software the list of parameters that were set to values different from the default ones. (DOCX 14 KB)

## References

[1] EFSA Panel on Plant Health (2014). Scientific opinion on pest categorisation of Grapevine Flavescence dorée. EFSA J.

[2] Weintraub PG, Beanland L (2005). Insect vectors of phytoplasmas. Annu Rev Entomol.

[3] Margaria P, Rosa C, Marzachì C, Turina M, Palmano S (2007). Detection of flavescence dorée phytoplasma in grapevine by reverse-transcription PCR. Plant Dis.

[4] Martini M, Murari M, Mori N, Bertaccini A (1999). Identification and epidemic distribution of two flavescence dorée—related phytoplasmas in Veneto (Italy). Plant Dis.

[5] Davis RE, Dally EL (2001). Revised subgroup classification of group 16SrV phytoplasmas and placement of flavescence dorée associated phytoplasmas in two distinct subgroups. Plant Dis.

[6] Arnaud G, Malembic-Maher S, Salar P, Bonnet P, Maixner M, Marcone C, Boudon-Padieu E, Foissac X (2007). Multilocus sequence typing confirms the close genetic interrelatedness of three distinct flavescence dorée phytoplasma strain clusters and group 16SrV phytoplasmas infecting grapevine and alder in Europe. Appl Environ Microbiol.

[7] Martini M, Botti S, Marcone C, Marzachì C, Casati P, Bianco PA, Benedetti R, Bertaccini A (2002). Genetic variability among flavescence dorée phytoplasmas from different origins in Italy and France. Mol Cell Probes.

[8] Belli G, Bianco PA, Conti M (2010). Grapevine yellows in Italy: past, present and future. J Plant Pathol.

[9] Albertazzi G, Milc J, Caffagni A, Francia E, Roncaglia E, Ferrari F, Tagliafico E, Stefani E, Pecchioni N (2009). Gene expression in grapevine cultivars in response to Bois Noir phytoplasma infection. Plant Sci.

[10] Hren M, Nikolic P, Rotter A, Blejec A, Terrier N, Ravnikar M, Dermastia M, Gruden K (2009). ‘Bois noir’ phytoplasma induces significant reprogramming of the leaf transcriptome in the field grown grapevine. BMC Genomics.

[11] Ehya F, Monavarfeshani A, Mohseni Fard E, Karimi Farsad L, Khayam Nekouei M, Mardi M, Salekdeh GH (2013). Phytoplasma-responsive microRNAs modulate hormonal, nutritional, and stress signalling pathways in Mexican lime trees. PLoS One.

[12] Mou H-Q, Lu J, Zhu S-F, Lin C-L, Tian G-Z, Xu X, Zhao W-J (2013). Transcriptomic analysis of Paulownia infected by Paulownia witches’-broom Phytoplasma. PLoS One.

[13] Liu LY, Tseng HI, Lin CP, Lin YY, Huang YH, Huang CK, Chang TH, Lin SS (2014). High-throughput transcriptome analysis of the leafy flower transition of Catharanthus roseus induced by peanut Witches’-broom phytoplasma infection. Plant Cell Physiol.

[14] Luge T, Kube M, Freiwald A, Meierhofer D, Seemüller E, Sauer S (2014). Transcriptomics assisted proteomic analysis of Nicotiana occidentalis infected by ‘Candidatus Phytoplasma mali’ strain AT. Proteomics.

[15] Monavarfeshani A, Mirzaei M, Sarhadi E, Amirkhani A, Khayam Nekouei M, Haynes PA, Mardi M, Salekdeh GH (2012). Shotgun proteomic analysis of the Mexican lime tree infected with ‘CandidatusPhytoplasma aurantifolia’. J Proteome Res.

[16] Gai YP, Li YQ, Guo FY, Yuan CZ, Mo YY, Zhang HL, Wang H, Ji XL (2014). Analysis of phytoplasma-responsive sRNAs provide insight into the pathogenic mechanisms of mulberry yellow dwarf disease. Sci Rep.

[17] Ji X, Gai Y, Lu B, Zheng C, Mu Z (2010). Shotgun proteomic analysis of mulberry dwarf phytoplasma. Proteome Sci.

[18] Siewert C, Luge T, Duduk B, Seemüller E, Büttner C, Sauer S, Kube M (2014). Analysis of expressed genes of the bacterium ‘Candidatus phytoplasma Mali’ highlights key features of virulence and metabolism. PLoS One.

[19] Oshima K, Ishii Y, Kakizawa S, Sugawara K, Neriya Y, Himeno M, Minato N, Miura C, Shiraishi T, Yamaji Y, Namba S (2011). Dramatic transcriptional changes in an intracellular parasite enable host switching between plant and insect. PLoS One.

[20] Carle P, Malembic-Maher S, Arricau-Bouvery N, Desqué D, Eveillard S, Carrère S, Foissac X (2011). Flavescence dorée phytoplasma genome: a metabolism oriented towards glycolysis and protein degradation. Bull Insectol (Supplement).

[21] Malembic-Maher S, Constable F, Cimerman A, Arnaud G, Carle P, Foissac X, Boudon-Padieu E (2008). A chromosome map of the Flavescence dorée phytoplasma. Microbiology.

[22] Kakizawa S, Oshima K, Jung HY, Suzuki S, Nishigawa H, Arashida R, Miyata S, Ugaki M, Kishino H, Namba S (2006). Positive selection acting on a surface membrane protein of the plant-pathogenic phytoplasmas. J Bacteriol.

[23] Boonrod K, Munteanu B, Jarausch B, Jarausch W, Krczal G (2012). An immunodominant membrane protein (Imp) of ‘Candidatus phytoplasma mali’ binds to plant actin. MPMI.

[24] Cimerman A, Pacifico D, Salar P, Marzachì C, Foissac X (2009). Striking diversity of vmp1, a variable gene encoding a putative membrane protein of the stolbur phytoplasma. Appl Environ Microbiol.

[25] Murolo S, Marcone C, Prota V, Garau R, Foissac X, Romanazzi G (2010). Genetic variability of the stolbur phytoplasma vmp1 gene in grapevines, bindweeds and vegetables. J Appl Microbiol.

[26] Schulthess B, Bloes DA, Francois P, Girard M, Schrenzel J, Bischoff M, Berger-Bachi B (2011). sigmaB-dependent yabJ-spoVG operon involved in the regulation of extracellular nuclease, lipase and protease expression in Staphylococcus aureus. J Bacteriol.

[27] Mastronunzio JE, Kurscheid S, Fikrig E (2012). Post-genomic analyses reveal development of infectious Anaplasma phagocytophilum during transmission from ticks to mice. J Bacteriol.

[28] Vannucci F, Foster D, Gebhart C (2013). Laser microdissection coupled with RNA-seq analysis of porcine enterocytes infected with an obligate intracellular pathogen (Lawsonia intracellularis). BMC Genomics.

[29] Darby AC, Armstrong SD, Bah GS, Kaur G, Hughes MA, Kay SM, Koldkjær P, Rainbow L, Radford AD, Blaxter ML, Tanya VN, Trees AJ, Cordaux R, Wastling JM, Makepeace BL (2012). Analysis of gene expression from the Wolbachia genome of a filarial nematode supports both metabolic and defensive roles within the symbiosis. Genome Res.

[30] Roggia C, Caciagli P, Galetto L, Pacifico D, Veratti F, Bosco D, Marzachì C (2014). Flavescence dorée phytoplasma titer in field-infected Barbera and Nebbiolo grapevines. Plant Pathol.

[31] Moran NA (1996). Accelerated evolution and Muller’s rachet in endosymbiotic bacteria. Proc Natl Acad Sci U S A.

[32] Bai X, Correa VR, Toruño TY, Ammar ED, Kamoun S, Hogenhout SA (2008). AY-WB phytoplasma secretes a protein that targets plant cell nuclei. MPMI.

[33] Sugio A, Kingdom HN, MacLean AM, Grieve VM, Hogenhout SA (2011). Phytoplasma protein effector SAP11 enhances insect vector reproduction by manipulating plant development and defense hormone biosynthesis. Proc Natl Acad Sci U S A.

[34] MacLean AM, Sugio A, Makarova OV, Findlay KC, Grieve VM, Tóth R, Nicolaisen M, Hogenhout SA (2011). Phytoplasma effector SAP54 induces indeterminate leaf-like flower development in Arabidopsis plants. Plant Physiol.

[35] Seemüller E, Sule S, Kube M, Jelkmann W, Schneider B (2013). The AAA+ ATPases and HflB/FtsH proteases of ‘Candidatus phytoplasma mali’: phylogenetic diversity, membrane topology, and relationship to strain virulence. MPMI.

[36] Miura C, Sugawara K, Neriya Y, Minato N, Keima T, Himeno M, Maejima K, Komatsu K, Yamaji Y, Oshima K, Namba S (2012). Functional characterization and gene expression profiling of superoxide dismutase from plant pathogenic phytoplasma. Gene.

[37] Bai X, Zhang J, Ewing A, Miller SA, Jancso Radek A, Shevchenko DV, Tsukerman K, Walunas T, Lapidus A, Campbell JW, Hogenhout SA (2006). Living with genome instability: the adaptation of phytoplasmas to diverse environments of their insect and plant hosts. J Bacteriol.

[38] Evans D, Marquez SM, Pace NR (2006). RNase P: interface of the RNA and protein worlds. Trends Biochem Sci.

[39] Tourasse NJ, Stabell FB, Reiter L, Kolstø AB (2005). Unusual group II introns in bacteria of the bacillus cereus group. J Bacteriol.

[40] Oshima K, Kakizawa S, Nishigawa H, Jung HY, Wei W, Suzuki S, Arashida R, Nakata D, Miyata S, Ugaki M, Namba S (2004). Reductive evolution suggested from the complete genome sequence of a plant-pathogenic phytoplasma. Nat Genet.

[41] Simon DM, Clarke NAC, McNeil BA, Johnson I, Pantuso D, Dai L, Chai D, Zimmerly S (2008). Group II introns in Eubacteria and Archaea: ORF-less introns and new varieties. RNA.

[42] Bonen L (1993). Trans-splicing of pre-mRNA in plants, animals, and protists. FASEB J.

[43] Cousineau B, Lawrence S, Smith D, Belfort M (2000). Retrotransposition of a bacterial group II intron. Nature.

[44] Del Campo M, Tijerina P, Bhaskaran H, Mohr S, Yang Q, Jankowsky E, Russell R, Lambowitz AM (2007). Do DEAD-Box proteins promote group II intron splicing without unwinding RNA?. Mol Cell.

[45] Toft C, Andersson SGE (2010). Evolutionary microbial genomics: insights into bacterial host adaptation. Nat Rev Genet.

[46] Leclercq S, Giraud I, Cordaux R (2011). Remarkable abundance and evolution of mobile group II introns in wolbachia bacterial endosymbionts. Mol Biol Evol.

[47] Margaria P, Turina M, Palmano S (2009). Detection of Flavescence dorée and Bois noir phytoplasmas, Grapevine leafroll associated virus-1 and -3 and Grapevine virus A from the same crude extract by reverse transcription-RealTime Taqman assays. Plant Pathol.

[48] Chang S, Puryear J, Cairney J (1993). A simple and efficient method for isolating RNA from pine trees. Plant Mol Biol Rep.

[49] Schneider B, Seemüller E, Smart CD, Kirkpatrick BC, Razin S, Tully JG (1995). Phylogenetic Classification of Plant Pathogenic Mycoplasma-Like Organisms or Phytoplasmas. Molecular and Diagnostic Procedures in Mycoplasmology.

[50] Lee IM, Gundersen DE, Hammond RW, Davis RE (1994). Use of mycoplasmalike organism (MLO) group-specific oligonucleotide primers for nested-PCR assays to detect mixed-MLO infections in a single host plant. Phytopathology.

[51] Bolger AM, Lohse M, Usadel B (2014). Trimmomatic: a flexible trimmer for Illumina sequence data. Bioinformatics.

[52] Carle P, Malembic-Maher S, Arricau-Bouvery N, Desqué D, Eveillard S, Carrère S, Foissac X (2011). ‘Flavescence dorée’ phytoplasma genome: a metabolism oriented towards glycolysis and protein degradation. Bull Insectol.

[53] Langmead B, Trapnell C, Pop M, Salzberg S (2009). Ultrafast and memory-efficient alignment of short DNA sequences to the human genome. Genome Biol.

[54] Trapnell C, Pachter L, Salzberg SL (2009). TopHat: discovering splice junctions with RNA-Seq. Bioinformatics.

[55] Grimplet J, Van Hemert J, Carbonell-Bejerano P, Dìaz-Riquelme J, Dickerson J, Fennell A, Pezzotti M, Martinez-Zapater J (2012). Comparative analysis of grapevine whole-genome gene predictions, functional annotation, categorization and integration of the predicted gene sequences. BMC Res Notes.

[56] Grabherr MG, Haas BJ, Yassour M, Levin JZ, Thompson DA, Amit I, Adiconis X, Fan L, Raychowdhury R, Zeng Q, Chen Z, Mauceli E, Hacohen N, Gnirke A, Rhind N, di Palma F, Birren BW, Nusbaum C, Lindblad-Toh K, Friedman N, Regev A (2011). Full-length transcriptome assembly from RNA-Seq data without a reference genome. Nat Biotech.

[57] Altschul SF, Gish W, Miller W, Myers EW, Lipman DJ (1990). Basic local alignment search tool. J Mol Biol.

[58] Huang X, Madan A (1999). CAP3: a DNA sequence assembly program. Genome Res.

[59] Dyrløv Bendtsen J, Nielsen H, von Heijne G, Brunak S (2004). Improved prediction of signal peptides: SignalP 3.0. J Mol Biol.

[60] Krogh A, Larsson BR, von Heijne G, Sonnhammer ELL (2001). Predicting transmembrane protein topology with a hidden markov model: application to complete genomes. J Mol Biol.

[61] Moriya Y, Itoh M, Okuda S, Yoshizawa AC, Kanehisa M (2007). KAAS: an automatic genome annotation and pathway reconstruction server. Nucl Acids Res.

[62] Conesa A, Götz S (2008). Blast2GO: a comprehensive suite for functional analysis in plant genomics. Int J Plant Genomics.

[63] Gambino G, Gribaudo I (2006). Simultaneous detection of nine grapevine viruses by multiplex reverse transcription-polymerase chain reaction with coamplification of a plant RNA as internal control. Phytopathology.

[64] Galetto L, Rashidi M, Yamchi A, Veratti F, Marzachì C, Bertaccini A (2014). In Vitro Expression of Phytoplasma Immunodominant Membrane Proteins. Phytoplasmas and Phytoplasma Diseases Management: how to Reduce Their Economic Impact.

[65] Osborn AM, Smith CJ (2005). Molecular Microbial Ecology.

[66] Gutha L, Casassa L, Harbertson J, Naidu R (2010). Modulation of flavonoid biosynthetic pathway genes and anthocyanins due to virus infection in grapevine (Vitis vinifera L.) leaves. BMC Plant Biol.

