# Supplementary material for: RNA-Seq profile of flavescence dorée phytoplasma in grapevine
Source: BMC Genomics. 2014 Dec 11;15(1):1088. doi: 10.1186/1471-2164-15-1088 (PMC4299374; doi:10.1186/1471-2164-15-1088)
Supplement: Supplementary file 1 — Additional file 1: Comparison among the three assembly strategies. Table reports: how the two libraries were used for the assembly (separated or in combination), the name of the resulting dataset, the datasets that were compared two-by-two at the end of each assembly approach, the number of assembled sequences for each dataset, the average length of sequences belonging to each dataset, the number of unique sequences for each dataset and the name of the dataset obtained after the merging with cap3. * and § indicate that FD-mapped reads and unmapped Vitis reads were used, respectively. (DOC 36 KB) [file 12864_2014_6831_MOESM1_ESM.doc]

|  | **libraries** | **Datasets of Trinity- assembled transcripts** | **Two-by-two compared datasets** | **Number of assembled sequences** | **Average length of sequences** | **Number of unique sequences** | **Datasets obtained after merging with cap3** |
| --- | --- | --- | --- | --- | --- | --- | --- |
| **1^st^ approach** | 120* | Dataset 1 | Dataset 1 | 233 | 257 bp | 28 | Dataset 3 |
|  | 120E* | Dataset 2 | Dataset 2 | 181 | 247 bp | 13 |  |
|  | | | | | | | |
| **2^nd^ approach** | 120* +  120E* | Dataset 4 | Dataset 4 | 347 | 287 bp | 127 | Dataset 5 |
|  | | | Dataset 3 | 303 | 262 bp | 0 |  |
|  | | | | | | | |
| **3^rd^ approach** | 120§ +  120E§ | Dataset 6 | Dataset 6 | 256 | 278 bp | 3 | Final FD transcriptome dataset |
|  | | | Dataset 5 | 347 | 288 bp | 114 |  |
