# Supplementary figures and images for: RNA-Seq profile of flavescence dorée phytoplasma in grapevine
Source: BMC Genomics. 2014 Dec 11;15(1):1088. doi: 10.1186/1471-2164-15-1088 (PMC4299374; doi:10.1186/1471-2164-15-1088)

## Slide 1
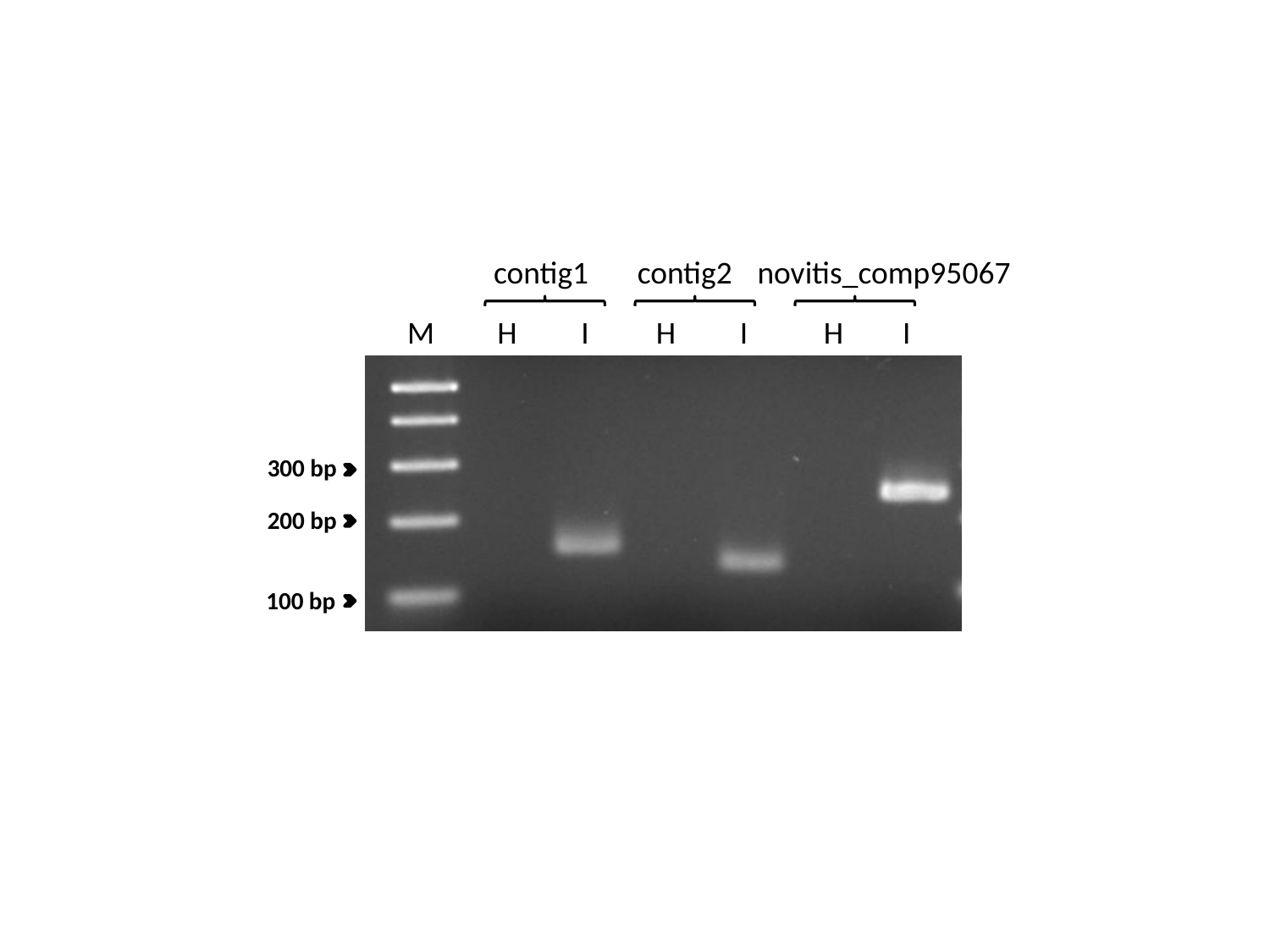

contig1
contig2
novitis_comp95067
M
H
I
H
I
H
I
300 bp
200 bp
100 bp

Supplement: Supplementary file 3 — Additional file 3: PCR products of the three RNA-Seq sequences with no match to the FD92 genome. Figure shows the amplicons obtained from healthy (H) and FD-C infected (I) periwinkle maintained in laboratory conditions. M = 1 kb plus marker (Thermo Fisher Scientific, Walthem, MA, USA).The primers used for amplifications were: contig1f 5′- GCCTGATAGAAAAAAAGTAG -3′ and contig1r 5′ – TTAGGAGAAATTTCTCCTGTAT - 3′ (Annealing temperature = 59°C); contig2f 5′- GAGAATCTGTAATGTATAAGG -3′and contig2r 5′ - TCAATATCTTCAGGAGTAGG - 3′(Annealing temperature = 60°C); novitis_comp95067f 5′- TGTGGCGATAACAAGAGCAA - 3′ and novitis_comp95067r 5′- TGTGCATAACCTTATCTCCTGC -3′ (Annealing temperature = 62°C). (PPTX 133 KB) [file 12864_2014_6831_MOESM3_ESM.pptx]
