# Supplementary material for: RNA-Seq profile of flavescence dorée phytoplasma in grapevine
Source: BMC Genomics. 2014 Dec 11;15(1):1088. doi: 10.1186/1471-2164-15-1088 (PMC4299374; doi:10.1186/1471-2164-15-1088)
Supplement: Supplementary file 4 — Additional file 4: Assembled transcripts that showed less than 100% identity at the nucleotide level with the FD92 annotated genes. Maltose/maltodextrin-binding periplasmic protein malE is present twice in the list because two assembled transcripts corresponded to two different parts of the corresponding FD92 gene. Most differences corresponded to non-synonymous substitutions, so these polymorphisms could potentially affect the protein conformation and/or functionality. *known/predicted membrane proteins; §ncRNA; TMD = transmembrane domain predicted by TMHMM. (DOCX 13 KB) [file 12864_2014_6831_MOESM4_ESM.docx]

| **RNA-Seq transcript ID** | **RNA-Seq transcript length** | **FD92 locus ID** | **Description** | **FD92 gene length** | **% identities** |
| --- | --- | --- | --- | --- | --- |
| Contig11 | 304 | vmpA-flado_0482_0026 * | Variable membrane protein A | 1146 | 95.2 |
| Contig187 | 979 | hflB-flado_0314_0026 * | ATP-dependent metallopeptidase | 1788 | 95.9 |
| Contig233 | 290 | grpE-flado_0031_0008 | HSP-70 cofactor | 900 | 97.9 |
| Contig17 | 207 | Ctg5389_0000012_0000263 * | Hypothetical protein with 1 TMD | 252 | 98.1 |
| Contig197 | 416 | malE-flado_8 084_0019 * | **Maltose/maltodextrin-binding periplasmic protein** | 1494 | 98.3 |
| Contig184 | 523 | vmpB-flado_0031_0011 * | Variable membrane protein B | 729 | 98.7 |
| Contig120 | 250 | Ctg0518_0000584_0003220 | Hypothetical N6 adenine-specific DNA methyltransferase | 2637 | 98.8 |
| Contig40 | 206 | Ctg8220_0017509_0018453 * | Hypothetical protein with 1 TMD | 945 | 99.5 |
| Contig140 | 232 | rpoC-flado_6343_0018 | DNA-directed RNA polymerase subunit beta' | 4077 | 99.6 |
| Contig9 | 283 | malE-flado_8084_0019 * | **Maltose/maltodextrin-binding periplasmic protein** | 1494 | 99.6 |
| Contig139 | 284 | malG-flado_8084_0020 * | Maltose transport system permease protein | 912 | 99.6 |
| Contig142 | 457 | infB-flado_5389_0003 | Translation initiation factor IF-2 | 1809 | 99.8 |
| Contig12^§^ | 698 |  | Group II catalytic intron | 698 | 98.6 |
