# Supplementary material for: RNA-Seq profile of flavescence dorée phytoplasma in grapevine
Source: BMC Genomics. 2014 Dec 11;15(1):1088. doi: 10.1186/1471-2164-15-1088 (PMC4299374; doi:10.1186/1471-2164-15-1088)
Supplement: Supplementary file 7 — Additional file 7: Parameters used for bioinformatics analysis. File reports for each software the list of parameters that were set to values different from the default ones. (DOCX 14 KB) [file 12864_2014_6831_MOESM7_ESM.docx]

**General hardware settings:** 40 CPU and RAM 150 GB.

**Parameters settings:**

- Trimmomatic: ILLUMINACLIP:TruSeq2-PE.fa:2:30:10 LEADING:3 TRAILING:3 SLIDINGWINDOW:4:15 MINLEN:36
- Bowtie for mapping reads to the whole FD92 genome: -X 300 (maximum insert size), -v 3 to include alignments with at most 3 mismatches.
- Bowtie for mapping reads either to predicted protein-coding genes of the FD92 genome or to sample 120 assembled transcripts: -X 300, –nofw or –norc (to distinguish reads mapped to the sense or the antisense strands, respectively).
- Cap3: “Overlap percent identity cutoff” set to 90; “Maximum gap length in any overlap” set to 5 and “Reverse orientation value” set to 0 to preserve the strand-specificity.
- Trinity: --SS_lib_type RF (to preserve the strand-specificity), --min_contig_length 150, --CuffFly --extended_lock (to reduce isoforms)
- Tophat: -r 153, –mate-std-dev 39, -i 20
